# The Minor Structural Difference between the Antioxidants Quercetin and 4′O-Methylquercetin Has a Major Impact on Their Selective Thiol Toxicity

**DOI:** 10.3390/ijms15057475

**Published:** 2014-04-30

**Authors:** Kristien J. A. Lemmens, Misha F. Vrolijk, Freek G. Bouwman, Wim J. F. van der Vijgh, Aalt Bast, Guido R. M. M. Haenen

**Affiliations:** 1 Department of Toxicology, Faculty of Health, Medicine and Life Sciences, Maastricht University, P.O. Box 616, Maastricht 6200 MD, The Netherlands; E-Mails: m.vrolijk@maastrichtuniversity.nl (M.F.V.); wjf.vandervijgh@maastrichtuniversity.nl (W.J.F.V.); a.bast@maastrichtuniversity.nl (A.B.); 2 Department of Human Biology, Faculty of Health, Medicine and Life Sciences, Maastricht University, P.O. Box 616, Maastricht 6200 MD, The Netherlands; E-Mail: freek.bouwman@maastrichtuniversity.nl

**Keywords:** quercetin, tamarixetin, thiol reactivity, creatine kinase, antioxidant

## Abstract

Antioxidants act as intermediates by picking up the high unselective reactivity of radicals and transferring it to other molecules. In this process the reactivity is reduced and becomes selective. This channeling of the reactivity can cause selective toxicity. The antioxidant quercetin is known to channel the reactivity towards thiol groups. The present study compares the thiol reactivity of quercetin with that of 4′O-methylquercetin (tamarixetin) towards creatine kinase (CK), a vital protein that contains a critical thiol moiety. Our results showed that oxidized quercetin and oxidized tamarixetin both adduct CK, which then loses its enzymatic function. Ascorbate, an important representative of the antioxidant network, is able to prevent adduction to and thus the inhibition of the enzyme by tamarixetin but not by quercetin. Apparently, tamarixetin is less thiol toxic than quercetin, because—rather than adduction to CK—tamarixetin quinone prefers to pass reactivity to the antioxidant network, *i.e*., to ascorbate. The findings exemplify that radical scavenging flavonoids pick up the reactivity of radicals and act as a pivot in directing the way the reactivity is channeled. A mere minor structural difference of only one methyl moiety between quercetin and tamarixetin appears to have a high impact on the selective, thiol toxicity.

## 1. Introduction

Flavonoids are polyphenols found in numerous fruits and vegetables having excellent antioxidant properties. After their discovery, the prevailing idea was that antioxidant intake was exclusively linked to health benefits. Antioxidants were thought to protect against radicals by simply scavenging them and thus fully annihilating their reactivity [1,2]. Contemporary research acknowledges this protective effect and append that by scavenging also reactive intermediates of the antioxidant are formed, which can be toxic [3].

Flavonoids are extraordinary potent antioxidants; this places them at the top of the pecking order meaning that flavonoids are first in line to scavenge reactive oxygen species (ROS). ROS take up an electron or hydrogen atom from the antioxidant flavonoid and this reduction converts the ROS into relatively harmless species. The oxidized flavonoids formed in the scavenging reaction are less reactive than the radical scavenged, which leads to selective reactivity. Oxidized flavonoids readily and specifically adduct thiol groups. Hence, oxidized flavonoids may threat vital cellular compounds containing a critical thiol group [4,5]. Apparently, a paradox is hidden in the functioning of an antioxidant flavonoid. Flavonoids protect against the reactivity of ROS but the other side of the coin is that during their protective effect, a potential thiol toxic product is formed.

The thiol containing enzyme creatine kinase (CK) is crucial for energy production in cells with a high energy turnover. Oxidative stress forms an onslaught on the energy level of the cell. Inhibition of CK aggravates the energy crisis which can finally lead to cell death [6]. This prompted us to examine thiol toxicity on CK.

The selective toxicity toward protein thiols might be circumvented when the reactivity of the oxidized flavonoid is absorbed by the antioxidant network of the cell. Ascorbate, an important representative in this network, can efficiently reduce an oxidized flavonoid and thus recycle the flavonoid. In this way the radical is neutralized by channeling its reactivity safely into the antioxidant network.

The flavonoids of interest to us are quercetin and the 4′O-methylquercetin, tamarixetin. Quercetin and tamarixetin are naturally occurring flavonoids which are present in our diet. Tamarixetin is also a metabolite of quercetin that is formed *in vivo* [7]. The cellular uptake of tamarixetin is higher than that of quercetin [8]. This supports a potential role of tamarixetin *in vivo*.

In this study the canalization of the reactivity of structurally related flavonoids, quercetin and tamarixetin, is unraveled at the level of protein reactivity because toxicity arises at this level. The preference of the oxidized flavonoids to direct their reactivity towards thiol containing proteins like CK or into the antioxidant network, specifically towards ascorbate, is determined. It is found that the minor difference in structure, *i.e*., the 4′O-methyl group, has a profound effect.

## 2. Results

### 2.1. Quercetin Quinone and Tamarixetin Quinone Inhibit Creatine Kinase

**Figure 1.**
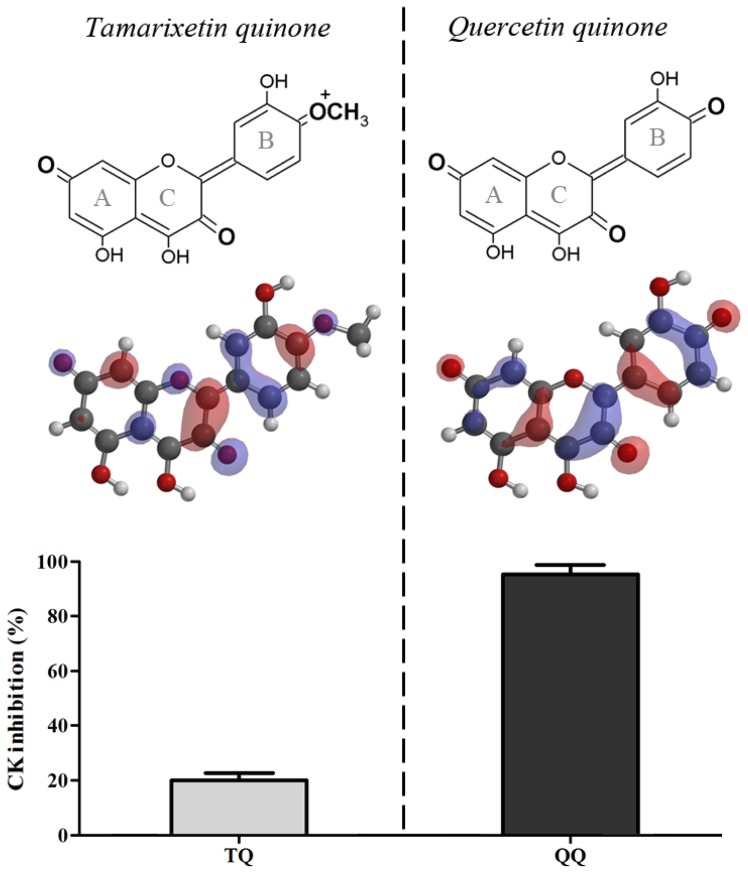
Structure and Lowest Unoccupied Molecular Orbital (LUMO) localization map of the preferred tautomer of quercetin quinone and tamarixetin quinone, and the effect of quercetin and tamarixetin oxidation on the enzyme activity of creatine kinase (CK). The carbonyl groups of quercetin quinone are positioned at maximal distance within the molecule and the LUMO is distributed over the phenolic rings, which explains why it behaves as a soft electrophile. Tamarixetin quinone has a positive charge and the LUMO is focused in the B-ring, which makes it a relatively hard electrophile. Quercetin and tamarixetin (50 μM) were oxidized by 50 μM H_2_O_2_ and 0.4 or 3.2 nM horseradish peroxidase (HRP), respectively, to obtain an equal rate of oxidation (5 μM/min). In the presence of 6.2 μM CK, the enzyme activity of CK was measured. Data are shown as mean ± SE (*n =* 4).

Quercetin quinone and tamarixetin quinone were generated *in situ* in an incubation mixture containing CK. Quercetin quinone was found to reduce CK activity (95%). Tamarixetin quinone also attenuated the activity of CK, but the extend of this reduction was less (20%) than that in the experiment with quercetin despite the equal rate of quinone formation (Figure 1).

**Figure 2.**
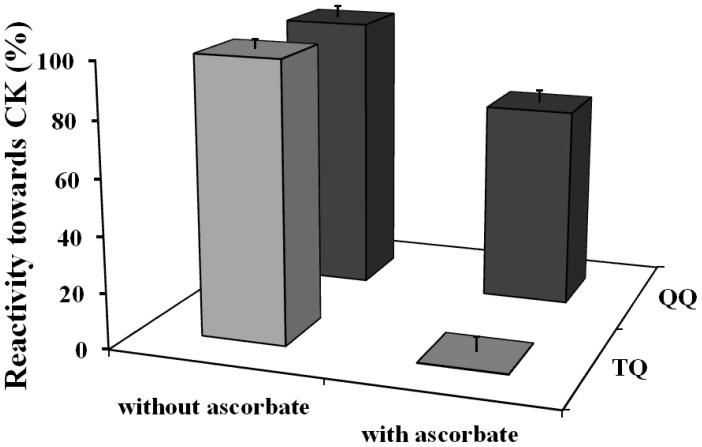
Effect of quercetin and tamarixetin oxidation on the enzyme activity of CK in presence of ascorbate. Quercetin and tamarixetin (50 μM) were oxidized by 50 μM H_2_O_2_ and HRP, at equal rate of oxidation (5 μM/min) in presence of 6.2 μM CK. The enzyme activity of CK was measured in the absence or presence of 50 μM ascorbate and expressed as percentage of the CK inhibition obtained without ascorbic acid. Data are shown as mean ± SE (*n =* 4).

Ascorbate only slightly protected against the inhibition of the activity caused by quercetin quinone (from 95% to 72%). In contrast, ascorbate completely protected the enzyme against the inactivation by the tamarixetin quinone, because the activity of CK was fully retained (Figure 2).

### 2.2. Quercetin Quinone and Tamarixetin Quinone Adduct Creatine Kinase

**Figure 3.**
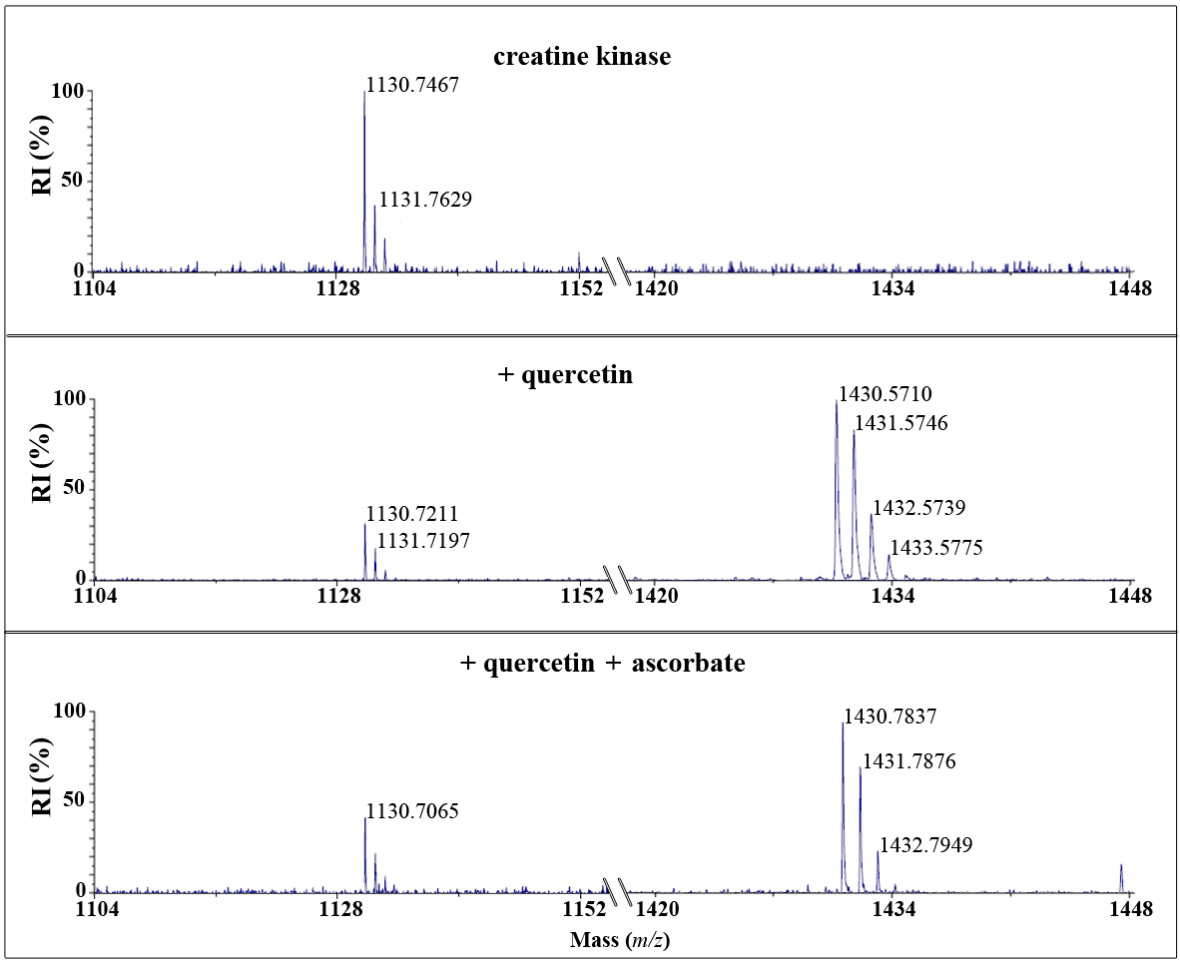
MALDI-ToF analysis of isolated creatine kinase (CK) (0.5 mg/mL) incubated with 50 μM quercetin, 0.4 nM HRP and 50 μM H_2_O_2_ with or without 50 μM ascorbate for 5 min at 37 °C. After trypsin digestion the mass spectrum of digested CK was measured. The control spectrum of CK displayed a peak at *m*/*z* 1130 and no peak at *m*/*z* 1430. The incubation with quercetin showed a peak at *m*/*z* 1430 which corresponds to the mass of the adduct of quercetin quinone (300 dalton) with the peptide having mass *m*/*z* 1130, whereas the peak at *m*/*z* 1130 decreased. The amino acid sequence of the peptide is GYTLPPHCSR, containing cysteine 146. The spectrum of CK incubated with quercetin in combination with ascorbate also showed a peak at *m*/*z* 1430. The peak at *m*/*z* 1130 was also present, but the relative intensity (RI) was less than the untreated CK.

**Figure 4.**
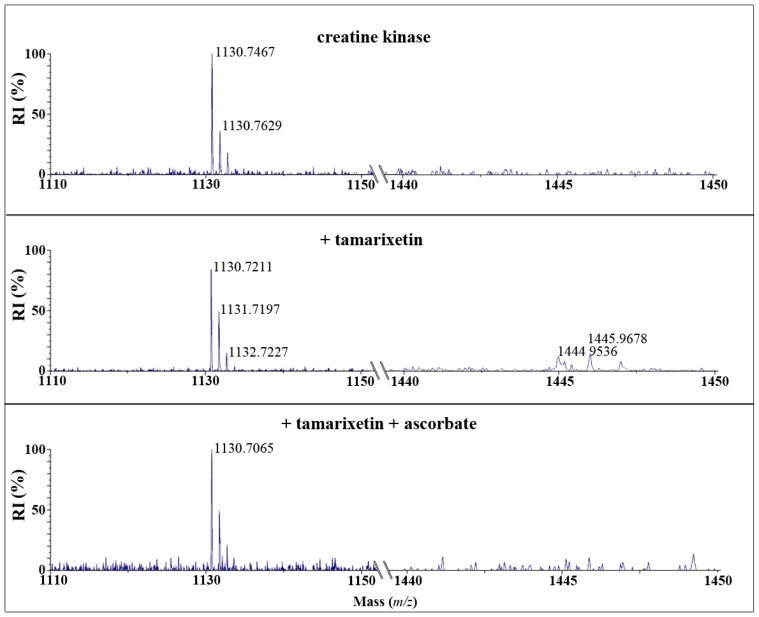
MALDI-ToF analysis of isolated creatine kinase (CK) (0.5 mg/mL) incubated with 50 μM tamarixetin, 3.2 nM HRP and 50 μM H_2_O_2_ with or without 50 μM ascorbate for 5 min at 37 °C. After trypsin digestion the mass spectrum of digested CK was measured. The control spectrum of CK displayed a peak at *m*/*z* 1130 and no peak at *m*/*z* 1444. The incubation with tamarixetin showed a peak at *m*/*z* 1444 which corresponds to the mass of the adduct of tamarixetin quinone (314 dalton) with the peptide having mass *m*/*z* 1130, whereas the peak at *m*/*z* 1130 decreased. The amino acid sequence of the peptide is GYTLPPHCSR, containing cysteine 146. The spectrum of CK incubated with tamarixetin in combination with ascorbate did not show a decrease in the intensity of the peak at *m*/*z* 1130 and an increase at *m*/*z* 1444.

After reaction of CK with quercetin quinone and tamarixetin quinone and subsequent trypsin digestion, MALDI-TOF analysis showed that the amount of the native peptide fragment-GYTLPPHCSR with a mass of *m*/*z* = 1130-was reduced. Fragments with a mass of *m*/*z* = 1430 or 1444 emerged after incubation with quercetin or tamarixetin, respectively (Figures 3 and 4). The increments of the mass of the fragment correspond to the molecular weight of the quinones, being 300 and 314 Da, respectively. Apparently, the flavonoid quinones adduct creatine kinase. The amount of adducted fragment formed was less after the reaction with tamarixetin quinone than after the reaction with quercetin quinone (Figures 3 and 4).

Ascorbate effectively prevented the formation of the flavonoid-protein fragment in the case of tamarixetin quinone but not in the case of quercetin quinone (Figures 3 and 4).

## 3. Discussion

Amongst antioxidants, flavonoids are at the top of the pecking order meaning that these antioxidants are first in line to react with radicals. Therefore, flavonoids can effectively protect against radical toxicity [9]. In this protection the flavonoids become oxidized. Due to their quinone structure, oxidized flavonoids react with nucleophilic thiol groups predominantly found in GSH and cysteine residues of proteins, resulting in selective thiol toxicity [4,10–13]. The toxicity of antioxidant flavonoids emerges when they adduct thiol groups of vital cellular proteins. Actually, it is not the flavonoid itself that displays this thiol toxicity but the oxidized form generated when the flavonoid exerts its antioxidant activity. The scavenging activity protects against the unselective reactivity of the radical, but can subsequently selectively induce toxicity to vital cellular compounds containing SH-groups. Ascorbic acid—an important antioxidant in the antioxidant network which has the potential to react with the oxidized flavonoids—might circumvent this thiol toxicity and as a bonus recycle the flavonoid [14].

In this study the reactivity of two structurally closely related flavonoids quercetin and its 4′O-methylated metabolite, tamarixetin have been investigated towards the thiol containing protein, creatine kinase (CK). It was found that the quinones of quercetin and tamarixetin are indeed thiol reactive. Our mass spectrometry data confirm adduction and indicate that both quinones react with cysteine 146 of CK, which is known to be essential for enzyme function [15]. The relative intensity of the adduct between CK and tamarixetin detected by MALDI-TOF was lower than that of quercetin and CK under the same experimental conditions. In accordance to these results, the activity of CK was less attenuated by tamarixetin quinone than quercetin quinone. This shows that the tamarixetin quinone is less thiol toxic than the quercetin quinone.

Ascorbate is able to efficiently protect against the inactivation of CK by oxidized tamarixetin. Remarkably, ascorbate only slightly protected against the inactivation of CK by the quercetin quinone. Apparently, the oxidized tamarixetin prefers to react with ascorbate instead of creatine kinase. Ascorbate is able to recycle the tamarixetin quinone to its parent flavonoid. The oxidized ascorbate formed in this redox reaction can, on its turn, be reduced, e.g., by dehydroascorbate reductase that uses NADH as cofactor. In this way the reactivity of the radical is completely neutralized, tamarixetin is recycled and the enzyme inactivation is prevented. These results show that the *O*-methylation on the 4′O position of quercetin ablates thiol toxicity.

The difference between quercetin and tamarixetin in directing the reactivity, originates from a minor change in the chemical structure and can be explained by Pearson’s HSAB (Hard and Soft Acids and Bases) concept [16]. Oxidized tamarixetin contains a positively charged group that has a relatively high polarity, which reflects in a high LUMO (Lowest Unoccupied Molecular Orbital) energy (21.33 kJ/mol) [17] (Figure 1). This makes oxidized tamarixetin a “hard” electrophile which prefers to react with ascorbate, a “hard” nucleophile. This is reflected in a high value of the competition between ascorbate and thiols (CAT), *i.e*., 14.5 ± 3.8 [17]. The CAT was determined by dividing the rate of reaction of the oxidized flavonoid with ascorbate by the rate of reaction of the oxidized flavonoid with the thiol GSH.

The quercetin quinone lacks this highly polar group and the carbonyl groups of oxidized quercetin are positioned at maximal distance. Therefore the LUMO of oxidized quercetin is distributed over all the phenolic rings which gives rise to a low LUMO energy (−112.14 kJ/mol) [17]. Consequently, oxidized quercetin is a relatively “soft” electrophile and will prefer to react with “soft” nucleophiles as the thiol group of GSH (the CAT of oxidized quercetin is 0.04 ± 0.03) [17] and as shown in the present study also with protein thiols.

## 4. Materials and Methods

### 4.1. Chemicals

Quercetin was purchased from Acros Organics (Geel, Belgium). Tamarixetin was obtained from Extrasynthese (Genay, France). Hydrogen peroxide (H_2_O_2_), l-ascorbic acid, horseradish peroxidase (HRP) and creatine kinase (CK) were obtained from Sigma (St. Louis, MO, USA). The creatine kinase kit was acquired from Bioo Scientific Corporation (Austin, TX, USA). Trypsin was purchased from Promega (Madison, WI, USA). Ammonium bicarbonate (ABC), acetonitrile (ACN), α-cyano-4-hydroxycinnamic acid and trifluoroacetic acid (TFA) were acquired from Sigma (St. Louis, MO, USA).

### 4.2. Creatine Kinase Activity

Quercetin and tamarixetin quinones were generated *in situ* by oxidizing the flavonoids (50 μM) with 50 μM H_2_O_2_ and 0.4 nM HRP in case of quercetin and 3.2 nM HRP in case of tamarixetin to obtain an equal rate of oxidation of the flavonoid (5 μM/min), in the presence of 6.2 μM CK in a 145 mM potassium-phosphate buffer pH 7.4. The influence of ascorbate was determined by adding 50 μM to the reaction mixture. The reactions were started by adding the HRP and carried out at 37 °C for 5 min.

Enzyme activity of creatine kinase (CK) in the reaction mixtures was measured by the catalytic conversion of ADP into ATP. Because ascorbate prevented the net consumption of tamarixetin, it was checked whether ascorbate did prevent the oxidation of tamarixetin. It was found that ascorbate did not inhibit the oxidation, but immediately reduced the oxidized tamarixetin (Supplementary Information). Hexokinase was used to convert ATP with glucose into glucose-6-phosphate. Finally, the glucose-6-phosphate formed converts NAD^+^ into NADH in the presence of glucose-6-phosphate dehydrogenase. The formation of NADH was measured spectrophotometrically at 340 nm during 5 min and reflects the CK activity.

### 4.3. Mass Spectrometry

In the presence of 6.2 μM CK in 50 mM ABC buffer pH 7.4, quercetin and tamarixetin (50 μM) were oxidized by 50 μM H_2_O_2_ and 0.4 or 3.2 nM HRP, respectively. The influence of ascorbate was determined by adding 50 μM to the reaction mixture. The oxidation reaction was carried out at 37 °C for 5 min. CK (0.5 mg/mL) was digested by adding 1 μg trypsin during 30 min at 37 °C. The digested samples were diluted 1:10 in 0.1% TFA. After dilution, 1 μL of the digest and 1 μL of matrix solution (2.5 mg/mL α-cyano-4-hydroxycinnamic acid in 50% ACN/0.1% TFA) were spotted on a 348-well-format target plate and air dried. Mass spectra were measured on the MALDI-TOF mass spectrometer (4800 MALDI-TOF analyzer; Applied Biosystems, Bridgewater, NJ, USA). The instrument was operated in positive reflector mode. Acquisition mass range was 800–3500 Da.

### 4.4. Statistics

All experiments were performed at least in triplicate. Data are expressed as means ± standard deviation (SD).

## 5. Conclusions

**Figure 5.**
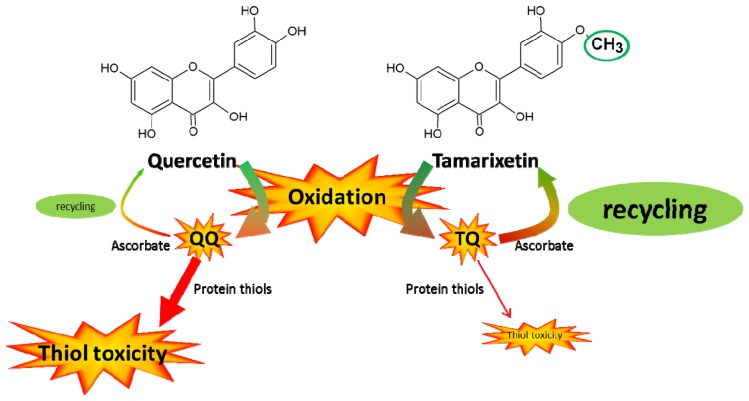
Overview of the difference in thiol toxicity between quercetin and tamarixetin. Tamarixetin and quercetin both protect against radical toxicity. The oxidized products that arise in this process (QQ and TQ) are thiol reactive. In the presence of ascorbate TQ preferentially passes its reactivity to ascorbate. An extra advantage is that ascorbate recycles TQ to the parent compound.

The present study addresses the selective toxicity of flavonoid antioxidants. Flavonoids pick up the reactivity of radicals and act as a pivot to channel the reactivity (Figure 5). Quercetin quinone channels its reactivity selectively towards thiol toxicity. The low but selective reactivity of oxidized antioxidants that directs the reactivity to a focal point (in this case thiols) has been implicated in the toxicity of antioxidants [18]. The selective reactivity of tamarixetin quinone directs the reactivity towards ascorbate in the antioxidant network. This might explain why tamarixetin is found to be less toxic than quercetin in cells [8]. Apparently, the introduction of only one methyl group in quercetin, giving tamarixetin, leads to a remarkably high reduction of the selective, thiol toxicity.

## Supplementary Information
