# Supplementary material for: The Minor Structural Difference between the Antioxidants Quercetin and 4′O-Methylquercetin Has a Major Impact on Their Selective Thiol Toxicity
Source: Int J Mol Sci. 2014 Apr 30;15(5):7475–84. doi: 10.3390/ijms15057475 (PMC4057684; doi:10.3390/ijms15057475)
Supplement: Supplementary file 1 [file ijms-15-07475-s001.pdf]

# Supplementary Information

Ascorbate prevented the net consumption of tamarixetin. The prevention of the oxidation of tamarixetin by ascorbate was checked.

## Materials and Methods

*Pectrophotometric Analysis*

Tamarixetin (50 µM) was oxidized by $H_2O_2$ (50 µM) and HRP (3.2 nM) in 145 mM potassium-phosphate buffer (pH 7.4). The incubation mixtures were analyzed spectrophotometrically in presence or absence of ascorbate (50 µM). The incubation mixture of ascorbate (50 µM) with (50 µM) and HRP (3.2 nM) in 145 mM potassium-phosphate buffer (pH 7.4) was also analyzed. All spectra were recorded from 220 to 600 nm. The UV/Vis scans were started 30, 90, 150, 210, 270 s after the addition of HRP.

## Results

*Spectrophotometric Analysis of the Oxidation of Tamarixetin*

The tamarixetin concentration decreased in the incubation mixture containing HRP/$H_2O_2$. Addition of 50 µM ascorbate to the incubation mixture shows no net consumption of tamarixetin (Figure S1A,B). HPLC analysis of the incubation mixtures confirmed that no tamarixetin was consumed in the presence of ascorbate (data not shown). In the incubation ascorbate was consumed, evidenced in the spectrum by the decrease of the absorption at 270 nm. When tamarixetin was omitted from the incubation mixture, the ascorbate concentration did not change (Figure S1C). For quercetin similar results have been reported [15].

## Conclusion

Ascorbate did not inhibit the oxidation, but immediately reduced the oxidized tamarixetin.

**Figure S1.** Spectrophotometrical analysis of the incubation mixture containing 50 µM tamarixetin, 50 µM $H_2O_2$ and 3.2 nM HRP (**A**); The same experiment was carried out in the presence of 50 µM ascorbate (**B**); Ascorbate was incubated with 50 µM $H_2O_2$ and 3.2 nM HRP (**C**); The UV/Vis scans were recorded 30, 90, 150, 210, 270 s after addition of HRP. The spectra of 50 µM $H_2O_2$ and 3.2 nM HRP, 50 µM tamarixetin and 50 µM ascorbate are shown (**D**).

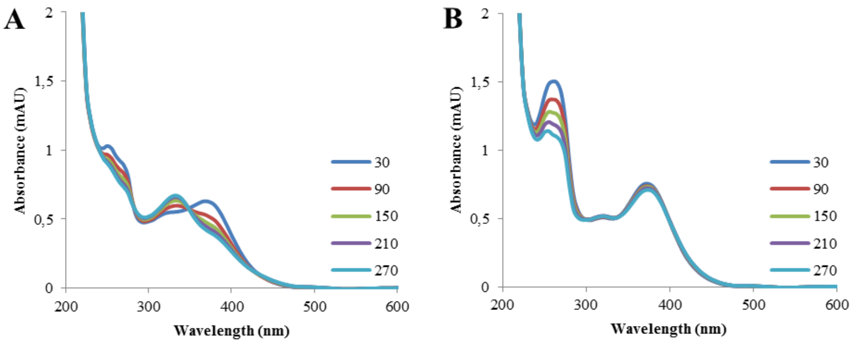

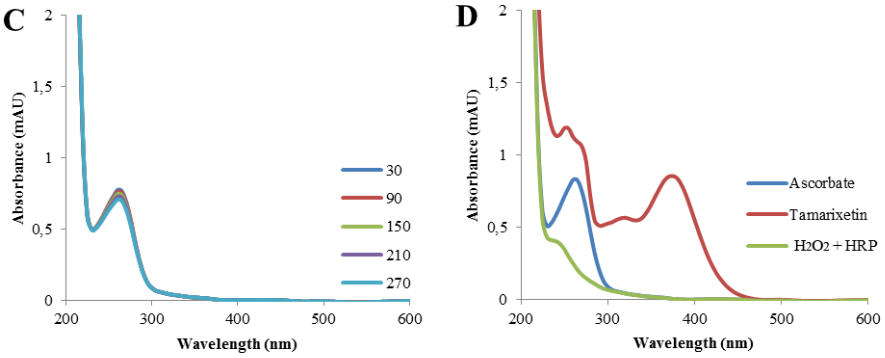


**Figure S1.** *Cont.*

© 2014 by the authors; licensee MDPI, Basel, Switzerland. This article is an open access article distributed under the terms and conditions of the Creative Commons Attribution license (http://creativecommons.org/licenses/by/3.0/).
